# Supplementary material for: Multi-objective de novo drug design with conditional graph generative model
Source: J Cheminform. 2018 Jul 24;10:33. doi: 10.1186/s13321-018-0287-6 (PMC6057868; doi:10.1186/s13321-018-0287-6)
Supplement: Supplementary file 1 — Additional file 1. Containing additional information about the implementation details of experiments. [file 13321_2018_287_MOESM1_ESM.pdf]

Supplementary Text for Multi-Objective De Novo Drug Design with Conditional Graph Generative Model

Yibo Li   Liangren Zhang*   Zhenming Liu*

Summary

1 Extracting structures from ChEMBL dataset **1**

2 Extracting scaffolds from drug dataset **1**

3 Training of activity models for JNK3 and GSK3β **2**

4 Algorithm for $q_\alpha(r|G)$ **2**

5 Additional proof 1 **3**

6 Additional proof 2 **4**

1 Extracting structures from ChEMBL dataset

Both graph based and SMILES based models are trained using structures extracted from ChEMBL. Molecule structures from ChEMBL[1] are first standardized using RDKit[2]. This process involves salt removal, molecule neutralization, removing isotopes, and conversion to canonical SMILES strings. We only keep molecules containing less than 50 heavy atoms and whose elements belong to the set $\{H, B, C, N, O, F, P, S, Cl, Br, I\}$. This result in a dataset containing 1.5 million molecules. 5-fold cross validation is performed during evaluation.

2 Extracting scaffolds from drug dataset

As discussed in the method part, the scaffold set S is extracted from the list of approved drugs in DrugBank[3]. Two type of structure is considered during extraction: (1) the Bemis-Murcko scaffolds, and (2) ring assemblies. The extraction process is performed using RDKit. Scaffolds with a molecular weight larger than 300 are removed. Fragments that are tautomer to each other are merged into a single entity, as they are unidentifiable during substructure matching

(since the matching algorithm in RDKit is design to ignore hydrogens). The resulted S contains a total of 1129 scaffold structures.

3 Training of activity models for JNK3 and GSK3β

For bioactivity dataset, search is performed on ExCAPE-DB[4] to extract activity data for each target. We use the activity flag provided by the database to separate the active and inactive compounds. This results in 3334 active compounds and 300186 inactive compounds for GSK3β, as well as 923 active compounds and 59412 inactive compounds for JNK3. For each dataset, 80% of data is randomly selected as training set, and the rest as test set. The RF models for each target are implemented using Scikit-learn[5] with the number of estimators (decision trees) set to 100. RDKit is used to calculate the ECFP6.

4 Algorithm for $q_\alpha(r|G)$

Algorithm 1 Sampling r from $q_\alpha(r|G)$ and get the likihood value

procedure SAMPLE(G, α) ▷ $G = (V, E)$
    Order atoms in V
    $G_{current} \leftarrow (\emptyset, \emptyset)$, $q \leftarrow 1$, $v \leftarrow$ null, $v_{stack} \leftarrow$ empty stack
    Mark all atoms and bonds in G unvisited
    while True **do**
        if $G_{current}$ is empty **then**
            $v* \leftarrow$ the atom in V with highest rank
            Sample *choise* from $Bernoulli(\alpha)$
            if $choise = 1$ **then**
                $v \leftarrow v*$, $q \leftarrow q \times \alpha$
            else
                $v \leftarrow$ randomly chosen atom from $V/v*$, $q \leftarrow q \times \frac{1-\alpha}{|V|-1}$
            end if
            Mark v visited
            $t \leftarrow$ the corresponding transition
            Append $(G_{current}, t)$ to r, set $G_{current} = t(G_{current})$
        else
            $V_e \leftarrow$ the set of visited atoms with unvisited connection to v
            if V_e is not empty **then**
                Sort V_e from newest visited to oldest visited
                for v_e in V_e **do**
                    $e \leftarrow$ the bond connecting v_e and v
                    Mark e visited
                    $t \leftarrow$ the corresponding transition
                    Append $(G_{current}, t)$ to r, set $G_{current} = t(G_{current})$
                end for

```
                V_n ← the set of unvisited neighbors of v
                if V_n is not empty then
                    v_stack.push(v)
                    v' ← v, v* ← the atom in V_n with highest rank
                    Sample choise from Bernoulli(α)
                    if choise = 1 then
                        v ← v*, q ← q × α
                    else
                        v ← randomly chosen atom in V_n/v*, q = q × (1−α)/(|V_n|−1)
                    end if
                    Mark v and the bond connecting v and v' visited
                    t ← the corresponding transition
                    Append (G_current, t) to r, set G_current = t(G_current)
                else
                    if v_stack is not empty then:
                        v ← v_stack.pop()
                        continue
                    else
                        t ← termination action
                        Append (G_current, t) to r
                        return r, q
                    end if
                end if
            end if
        end if
    end while
end procedure
```

5 Additional proof 1

Here, we demonstrate that druing the training of conditional generative model, the follwing objective is being minimized:

$$L(\boldsymbol{\theta}) = \mathbb{E}_{\mathbf{c}\sim p(\mathbf{c})}[D_{KL}(p(\mathbf{x}|\mathbf{c})||q_{\boldsymbol{\theta}})] \tag{1}$$

We can rewrite $L(\boldsymbol{\theta})$ as follows:

$$\begin{aligned} L(\boldsymbol{\theta}) &= \mathbb{E}_{\mathbf{c}\sim p(\mathbf{c})}[D_{KL}(p(\mathbf{x}|\mathbf{c})||q_{\boldsymbol{\theta}})] \\ &= \mathbb{E}_{\mathbf{c}\sim p(\mathbf{c})}[\mathbb{E}_{\mathbf{x}\sim p(\mathbf{x}|\mathbf{c})}[\log p(\mathbf{x}|\mathbf{c}) - \log q_{\boldsymbol{\theta}}(\mathbf{x})]] \\ &= \mathbb{E}_{(\mathbf{c},\mathbf{x})\sim p}[\log p(\mathbf{x}|\mathbf{c})] - \mathbb{E}_{(\mathbf{c},\mathbf{x})\sim p}[\log q_{\boldsymbol{\theta}}(\mathbf{x}|\mathbf{c})] \end{aligned} \tag{2}$$

$\mathbb{E}_{(\mathbf{c},\mathbf{x})\sim p}[\log p(\mathbf{x}|\mathbf{c})]$ is in fact a constant term with respect to $\boldsymbol{\theta}$. Therefore, it can be safely omitted from $L(\boldsymbol{\theta})$:

$$L(\boldsymbol{\theta}) = -\mathbb{E}_{(\mathbf{c},\mathbf{x})\sim p}[\log q_{\boldsymbol{\theta}}(\mathbf{x}|\mathbf{c})] \tag{3}$$

This value can be approximated using Monte Carlo sampling:

$$\hat{L}(\boldsymbol{\theta}) = -\frac{1}{N}\sum_{i=1}^{N} \log q_{\boldsymbol{\theta}}(\mathbf{x}_i|\mathbf{c}_i) \tag{4}$$

Where $(\mathbf{c}_1, \mathbf{x}_1), ..., (\mathbf{c}_N, \mathbf{x}_N)$ are sampled from the data distribution $p(\mathbf{c}, \mathbf{x})$. It is easy to see that eq. 4 is exactly the negative log-likelihood (NLL) loss used in MLE based training of conditional generative models.

6 Additional proof 2

Here, we show that optimizing the objective function $G(\mathbf{x})$ in the REINVENT method is equivalent to optimizing the KL divergence $D_{KL}(q_{\boldsymbol{\theta}}||p(\mathbf{c}|\mathbf{x}))$. In fact, if we set the score function $\sigma S(\mathbf{x})$ to $\log p(\mathbf{c}|\mathbf{x})$, it can be proved that:

$$\nabla_{\boldsymbol{\theta}} D_{KL}(q_{\boldsymbol{\theta}}||p(\mathbf{x}|\mathbf{c})) = -\mathbb{E}_{\mathbf{x}\sim q_{\boldsymbol{\theta}}}[\nabla_{\boldsymbol{\theta}} G(\mathbf{x})] \tag{5}$$

We expand the term $\nabla_{\boldsymbol{\theta}} D_{KL}(q_{\boldsymbol{\theta}}||p(\mathbf{x}|\mathbf{c}))$ as follows:

$$\begin{aligned}\nabla_{\boldsymbol{\theta}} D_{KL}(q_{\boldsymbol{\theta}}||p(\mathbf{x}|\mathbf{c})) &= \nabla_{\boldsymbol{\theta}} \mathbb{E}_{\mathbf{x}\sim q_{\boldsymbol{\theta}}}[\log q_{\boldsymbol{\theta}}(\mathbf{x}) - \log p(\mathbf{x}|\mathbf{c})] \\ &= \nabla_{\boldsymbol{\theta}} \mathbb{E}_{\mathbf{x}\sim q_{\boldsymbol{\theta}}}[\log q_{\boldsymbol{\theta}}(\mathbf{x}) - \log p(\mathbf{x}) - \log p(\mathbf{c}|\mathbf{x})] \\ &= \mathbb{E}_{\mathbf{x}\sim q_{\boldsymbol{\theta}}}[(\log q_{\boldsymbol{\theta}}(\mathbf{x}) - \log p(\mathbf{x}) - \log p(\mathbf{c}|\mathbf{x}))\nabla_{\boldsymbol{\theta}} \log q_{\boldsymbol{\theta}}(\mathbf{x})] \\ &= \mathbb{E}_{\mathbf{x}\sim q_{\boldsymbol{\theta}}}[\nabla_{\boldsymbol{\theta}}(\log q_{\boldsymbol{\theta}}(\mathbf{x}) - \log p(\mathbf{x}) - \log p(\mathbf{c}|\mathbf{x}))^2]\end{aligned} \tag{6}$$

Since we have $G(\mathbf{x}) = -(\log p(\mathbf{x}) + \log p(\mathbf{c}|\mathbf{x}) - \log q_{\boldsymbol{\theta}}(\mathbf{x}))^2$, we can get the following equivalence:

$$\nabla_{\boldsymbol{\theta}} D_{KL}(q_{\boldsymbol{\theta}}||p(\mathbf{x}|\mathbf{c})) = -\mathbb{E}_{\mathbf{x}\sim q_{\boldsymbol{\theta}}}[\nabla_{\boldsymbol{\theta}} G(\mathbf{x})] \tag{7}$$

References

[1] Gaulton, A., Bellis, L.J., Bento, A.P., Chambers, J., Davies, M., Hersey, A., Light, Y., McGlinchey, S., Michalovich, D., Al-Lazikani, B.: Chembl: a large-scale bioactivity database for drug discovery. Nucleic Acids Res **40**(D1), 1100–1107 (2011)

[2] RDKit: Open Source Cheminformatics. `http://www.rdkit.org/`

[3] Wishart, D.S., Knox, C., Guo, A.C., Shrivastava, S., Hassanali, M., Stothard, P., Chang, Z., Woolsey, J.: Drugbank: a comprehensive resource for in silico drug discovery and exploration. Nucleic Acids Res **34**(Database issue), 668–672 (2006)

[4] Sun, J., Jeliazkova, N., Chupakhin, V., Golib-Dzib, J.-F., Engkvist, O., Carlsson, L., Wegner, J., Ceulemans, H., Georgiev, I., Jeliazkov, V.: Excape-db: an integrated large scale dataset facilitating big data analysis in chemogenomics. J Cheminform **9**(1), 17 (2017)

[5] Pedregosa, F., Varoquaux, G., Gramfort, A., Michel, V., Thirion, B., Grisel, O., Blondel, M., Prettenhofer, P., Weiss, R., Dubourg, V.: Scikit-learn: Machine learning in python. J Mach Learn Res **12**(Oct), 2825–2830 (2011)
