# Supplementary material for: Multi-objective de novo drug design with conditional graph generative model
Source: J Cheminform. 2018 Jul 24;10:33. doi: 10.1186/s13321-018-0287-6 (PMC6057868; doi:10.1186/s13321-018-0287-6)
Supplement: Supplementary file 2 — Additional file 2. Contianing supplementary figures. [file 13321_2018_287_MOESM2_ESM.pdf]

Supplementary Figures for Multi-Objective De Novo Drug Design with Conditional Graph Generative Model

Yibo Li Zhenming Liu Liangren Zhang*

List of Figures

S1 Decoding process 2
S2 Decoding process in detail 3
S3 Number of decoding steps requried for SMILES based and graph based model 4
S4 ROC curves 5

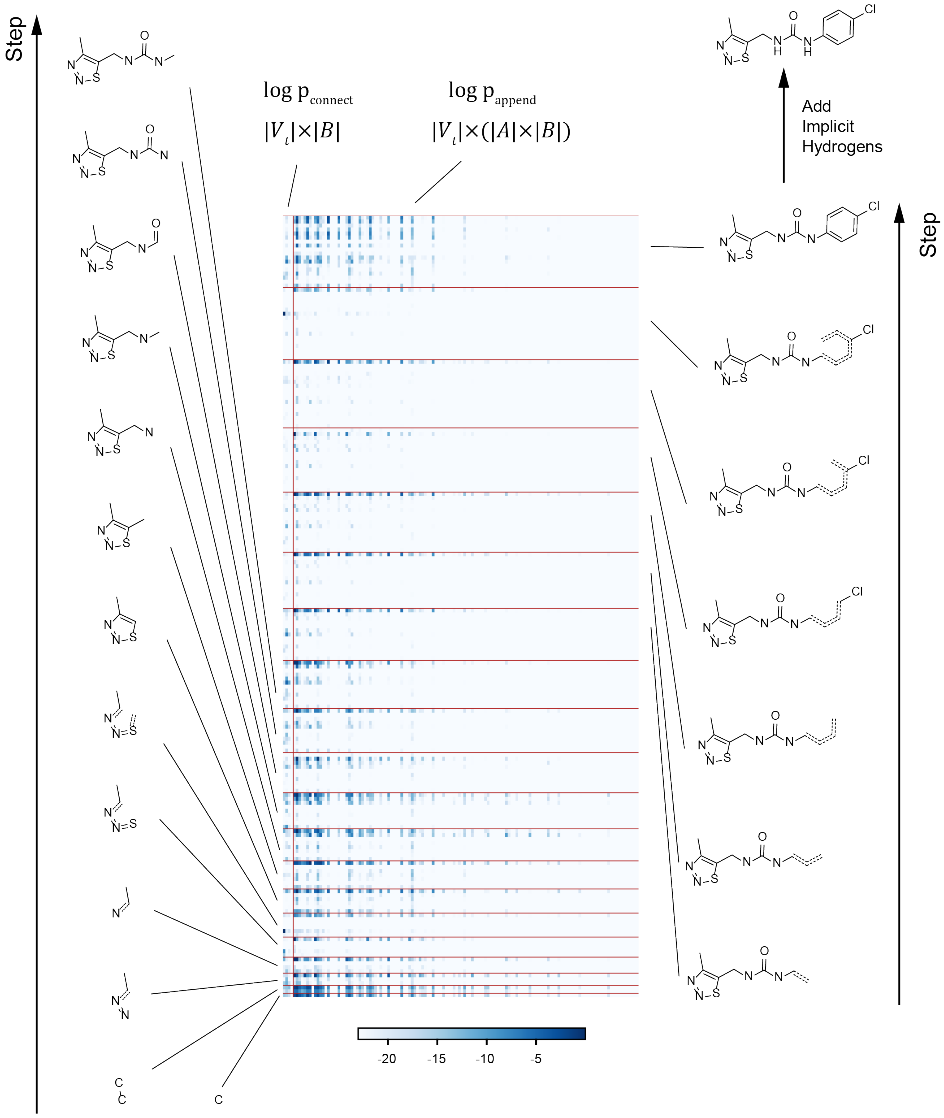

Figure S1: The decoding process of graph based generator. The molecule used in this example is randomly sampled from the graph generator. Structures at two sides indicates G_i at each step. The matrix in the center denote the probability distribution transition actions, where horizontal lines separate different steps, and the vertical line separates connection actions and append actions. Probability of termination at each step is not shown in this figure.

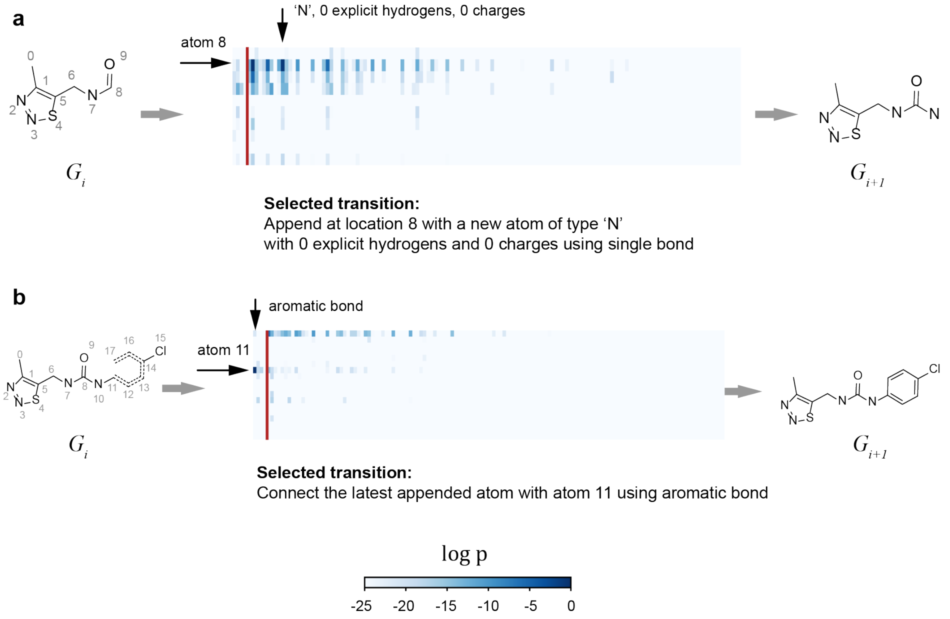

Figure S2: Detailed action performed at step 11 and step 19 during the decoding of the molecule in Figure S1.

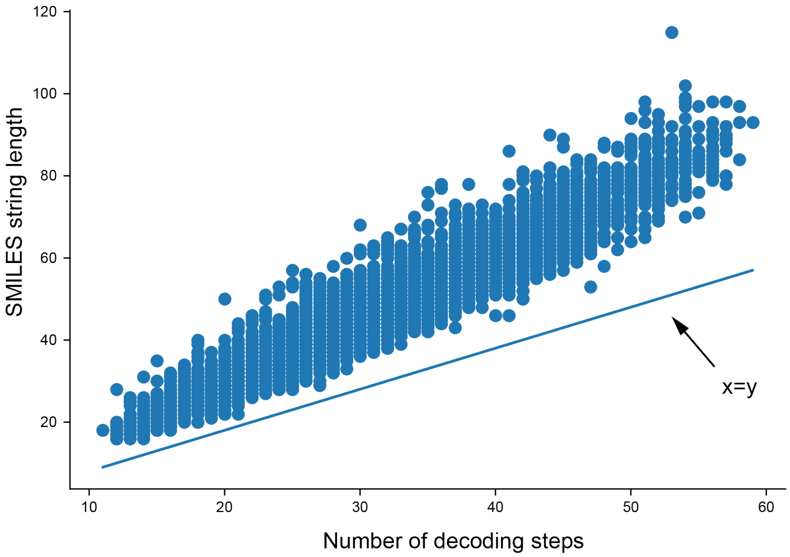

Figure S3: Relationship between the number of decoding steps required for a given molecule and the length of the corresponding SMILES. It can be shown that graph based decoding generally requires less steps compared with SMILES based decoding.

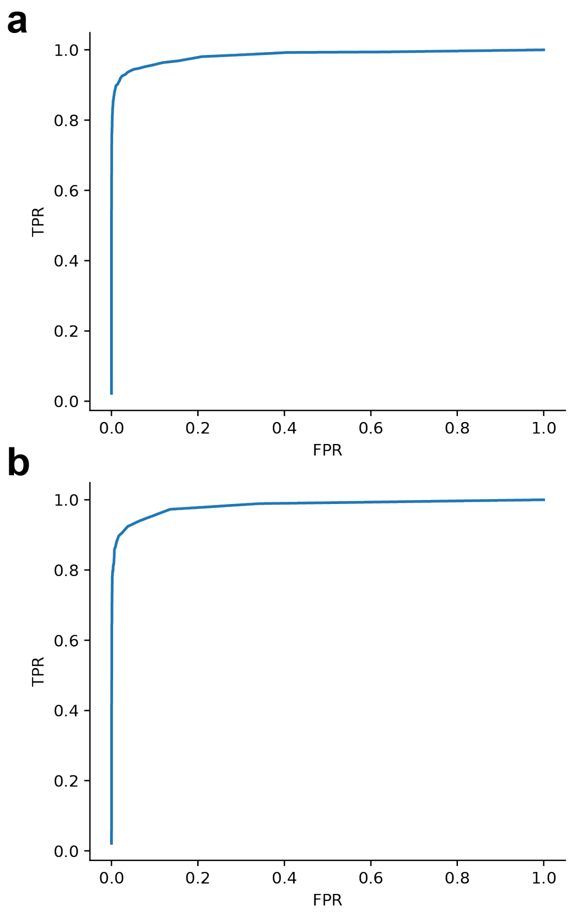

Figure S4: ROC curves for the predictive model of **a**. JNK3 and **b**. GSK3β
